# An extracellular *Staphylococcus epidermidis* polysaccharide: relation to Polysaccharide Intercellular Adhesin and its implication in phagocytosis

**DOI:** 10.1186/1471-2180-12-76

**Published:** 2012-05-17

**Authors:** Anastasia I Spiliopoulou, Maria I Krevvata, Fevronia Kolonitsiou, Llinos G Harris, Thomas S Wilkinson, Angharad P Davies, Georgios O Dimitracopoulos, Nikos K Karamanos, Dietrich Mack, Evangelos D Anastassiou

**Affiliations:** 1 Department of Microbiology, School of Medicine, University of Patras, Patras, Greece; 2 Medical Microbiology and Infectious Diseases, Institute of Life Science, The College of Medicine, Swansea University, Swansea, UK; 3 Laboratory of Biochemistry, Department of Chemistry, University of Patras, Patras, Greece

## Abstract

**Background:**

The skin commensal and opportunistic pathogen *Staphylococcus epidermidis* is a leading cause of hospital-acquired and biomaterial-associated infections. The polysaccharide intercellular adhesin (PIA), a homoglycan composed of β-1,6-linked N-acetylglucosamine residues, synthesized by enzymes encoded in *icaADBC* is a major functional factor in biofilm accumulation, promoting virulence in experimental biomaterial-associated *S. epidermidis* infection. Extracellular mucous layer extracts of *S. epidermidis* contain another major polysaccharide, referred to as 20-kDa polysaccharide (20-kDaPS), composed mainly out of glucose, N-acetylglucosamine, and being partially sulfated. 20-kDaPS antiserum prevents adhesion of *S*. *epidermidis* on endothelial cells and development of experimental keratitis in rabbits. Here we provide experimental evidence that 20-kDaPS and PIA represent distinct molecules and that 20-kDaPS is implicated in endocytosis of *S*. *epidermidis* bacterial cells by human monocyte-derived macrophages.

**Results:**

Analysis of 75 clinical coagulase-negative staphylococci from blood-cultures and central venous catheter tips indicated that 20-kDaPS is expressed exclusively in *S. epidermidis* but not in other coagulase-negative staphylococcal species. Tn*917*-insertion in various locations in *icaADBC* in mutants M10, M22, M23, and M24 of *S. epidermidis* 1457 are abolished for PIA synthesis, while 20-kDaPS expression appears unaltered as compared to wild-type strains using specific anti-PIA and anti-20-kDaPS antisera. While periodate oxidation and dispersin B treatments abolish immuno-reactivity and intercellular adhesive properties of PIA, no abrogative activity is exerted towards 20-kDaPS immunochemical reactivity following these treatments. PIA polysaccharide I-containing fractions eluting from Q-Sepharose were devoid of detectable 20-kDaPS using specific ELISA. Preincubation of non-20-kDaPS-producing clinical strain with increasing amounts of 20-kDaPS inhibits endocytosis by human macrophages, whereas, preincubation of 20-kDaPS-producing strain ATCC35983 with 20-kDaPS antiserum enhances bacterial endocytosis by human macrophages.

**Conclusions:**

In conclusion, *icaADBC* is not involved in 20-kDaPS synthesis, while the chemical and chromatographic properties of PIA and 20-kDaPS are distinct. 20-kDaPS exhibits anti-phagocytic properties, whereas, 20-kDaPS antiserum may have a beneficial effect on combating infection by 20-kDaPS-producing *S. epidermidis*.

## Background

*Staphylococcus epidermidis* and other coagulase-negative staphylococci (CoNS) constitute the most frequent causes of hospital-acquired infections and are often associated with the use of medical devices [1]. Virulence is mainly attributed to surface colonization and biofilm formation [2]. A biofilm represents an adherent, structured, high density community of bacterial cells [3] embedded in an extracellular matrix, previously called slime. Polysaccharide Intercellular Adhesin (PIA), a homoglycan composed of β-1,6-linked 2-deoxy-2-amino-D-glucopyranosyl residues, is considered to be the major functional component mediating intercellular adhesion in *S. epidermidis* biofilms [4-7]. Biofilm formation mediated by PIA is a major virulence factor in experimental biomaterial-associated infection [8] and provides also protection against opsonophagocytosis and activity of anti-microbial peptides [9,10]. The genes encoding PIA production are organized in the *icaADBC* operon [11-13].

Moreover, a polysaccharide molecule with 20-kDa average molecular mass, defined as 20-kDaPS, was isolated from *S. epidermidis* ATCC35983 (RP12), ATCC35984 (RP62A) and clinical biofilm-producing strains by ion-exchange chromatography and gel filtration [14-16]. Its purity, charge density and molecular integrity have been confirmed by reverse polarity capillary electrophoresis [16]. 20-kDaPS consists mainly of glucose and N-acetylglucosamine, and is partially sulfated. Proposed structure of 20-kDaPS is 30–35 molecules of glucose, 1–3 molecules of xylose and fucose, 61–65 molecules of glucosamine (6–7 N-sulfated) (also perhaps N- acetyl- and/or succinated) and 3–4 molecules of glucuronic acid [14]. This polysaccharide represents 60-65% of total slime carbohydrate and seems to be one of the main antigenic components of slime [17,18]. Immunization of rabbits with purified 20-kDaPS elicits production of antibodies reacting specifically with 20-kDaPS and biofilm-producing reference strain ATCC35983 (RP12) and other biofilm-producing clinical *S. epidermidis* strains, but not with other CoNS or *S. aureus* clinical isolates [19]. Protective value of 20-kDaPS antibodies has been proven in experimental keratitis protocols, where passive and active immunization of rabbits with 20-kDaPS antigen and anti-20-kDaPS exhibit beneficial properties [20-22]. Administration of intravenous immunoglobulin preparations with high anti-20-kDaPS titers in preterm neonates reduces risk of bacteraemia caused by biofilm-producing *S. epidermidis*[23]. Finally, experimental data suggest that 20-kDaPS is associated with attachment of *S*. *epidermidis* to endothelial cells [24].

 Several other polysaccharide molecules have been associated with biofilm accumulation or initial adherence on surfaces, such as PS/A (Capsular Polysaccharide Adhesin) or PNSG (Poly N-Succinyl Glucosamine), finally defined as PNAG [25-28], and SAA (Slime Associated Antigen) [29,30]. As other polysaccharide molecules associated with *S*. *epidermidis*’ pathogenesis turned out to be identical or related to PIA [31-36], the aim of this study was to define the relation of 20-kDaPS and PIA using isogenic mutants with Tn*917*-insertions in various locations in *icaADBC*, specific antisera and specific glycosidase and chemical treatments. In addition, *in vitro* experiments were conducted exploring 20-kDaPS biological interference in phagocytosis by human macrophages.

## Results

### Detection of 20-kDaPS, PIA expression and *icaADBC*-genotype in clinical CoNS isolates

Among fifty (50) clinical *S. epidermidis* strains, eighteen (36%) were found *ica*^**+**^ biofilm^**+**^ 20-kDaPS^**+**^, ten (20%) *ica*^**-**^ biofilm^**-**^ 20-kDaPS^**-**^, six (12%) *ica*^+^ biofilm^-^ 20-kDaPS^+^, six (12%) *ica*^**-**^ biofilm^**-**^ 20-kDaPS^**+**^, five (10%) *ica*^**+**^ biofilm^**-**^ 20-kDaPS^**-**^ and five (10%) strains *ica*^**+**^ biofilm^**+**^ 20-kDaPS^**-**^. All other CoNS (n = 25) were *ica*^-^ biofilm^-^ 20-kDaPS^-^. All *ica*^+^ biofilm^+^*S. epidermidis* strains were PIA-positive by specific immunofluorescence test, whereas, *ica*^**-**^ biofilm^-^ or *ica*^**+**^ biofilm^**-**^ strains were PIA-negative. In our *S. epidermidis* strain collection, 46% (n = 23) were PIA positive and 60% (n = 30) were 20-kDaPS positive. *IcaADBC* prevalence in our collection was 68%, whereas 46% of *S. epidermidis* strains were biofilm-producing. 20-kDaPS expression among *ica*^+^*S. epidermidis* strains was 70% (24 *ica*^+^ 20-kDaPS^+^ amongst 34 *ica*^+^*S. epidermidis* strains), whereas, 20-kDaPS expression among *ica*^-^ strains was 37% (6 *ica*^**-**^ 20-kDaPS^**+**^ amongst 16 *ica*^**-**^*S. epidermidis* strains). 20-kDaPS expression in relation to biofilm formation reveals that 78% of biofilm-producing *S. epidermidis* strains expressed 20-kDaPS (18 biofilm^**+**^ 20-kDaPS^**+**^ in 23 biofilm^**+**^*S. epidermidis* strains), whereas, 44% of biofilm-negative strains were 20-kDaPS positive (12 biofilm^-^ 20-kDaPS^+^ of 27 biofilm^-^*S. epidermidis* strains). These results show that the majority of clinical *S. epidermidis* isolates express 20-kDaPS and that there is no strict correlation of *icaADBC*-genotype or biofilm phenotype and expression of 20-kDaPS.

### Expression of 20-kDaPS and PIA by *S*. *epidermidis* strains with known genetic backgrounds

**Figure 1:**
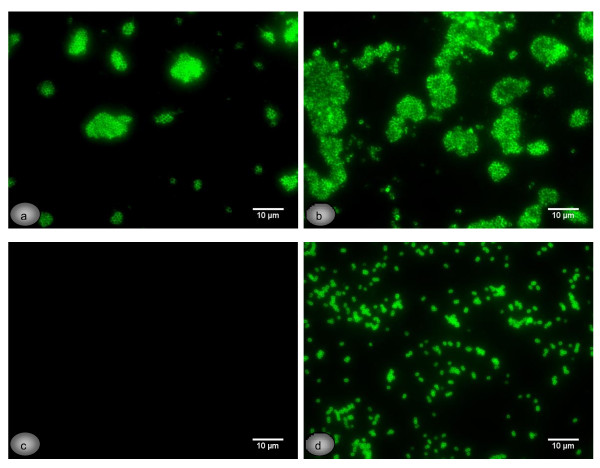
**Immunofluorescence detection of PIA and 20-kDaPS on reference strains.** Immunofluorescence detection of PIA (**a**, **c**) and 20-kDaPS (**b**, **d**) on *S*. *epidermidis* 1457 (**a**, **b**) and *icaA*-insertion mutant *S. epidermidis* 1457-M10 (**c**, **d**), grown in TSB medium, utilizing PIA and 20-kDaPS specific rabbit antisera, respectively.

**Figure 2:**
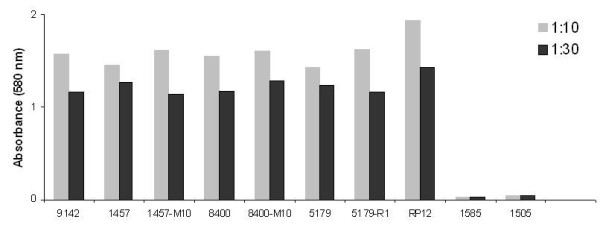
**20-kDaPS expression in reference strains.** Microtiter plates were coated with bacterial suspensions (absorbance_578_ =1.0) diluted 1:10 and 1:30, respectively, in PBS and incubated with 20-kDaPS antiserum at a 1:3,000 dilution. Results represent mean absorbance values ± SDs for two independent experiments performed in triplicate.

**Figure 3:**
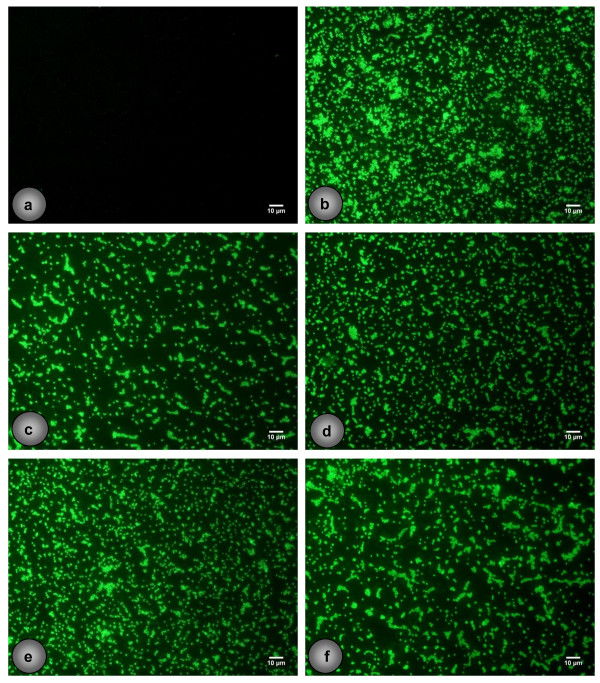
**Immunofluorescence detection of 20-kDaPS on selected strains.** Immunofluorescence detection of 20-kDaPS on *S. epidermidis* (**a**) 1505, (**b**) 1457, (**c**) 1457-M10, (**d**) M22, (**e**) M23 and (**f**) M24. Scale bar stands for 10 μm.

Using an indirect immunofluorescence test with specific anti-PIA antiserum *S. epidermidis* strains 1457, 8400, and 9142 were shown to express PIA, while the isogenic *icaA*-insertion mutants 1457-M10, M24 and 8400-M10 and isogenic *icaC*-insertion mutants M22 and M23 did not express PIA. Similarly, *S. epidermidis* 5179, 5179R1 and 1585 did not synthesize PIA as in the former two strains *icaADBC* is inactivated through insertion of IS*257*[37], while 1585 is *icaADBC*-negative. Using specific anti-20-kDaPS antiserum *S. epidermidis* 1457, 1457-M10, M22, M23, M24, 8400, 8400-M10, 9142, 5179, 5179R1 were 20-kDaPS positive, whereas, *S. epidermidis* strain 1585 was 20-kDaPS negative. A representative immunofluorescence test with anti-PIA and anti-20-kDaPS antisera, comparing *S. epidermidis* 1457 and 1457-M10, is displayed in Figure 1. An identical expression pattern of 20-kDaPS was independently demonstrated for these strains using specific ELISA, excluding that there are significant quantitative differences in 20-kDaPS antigen expression between the isogenic mutant strain pairs (Figure 2). 20-kDaPS detection in transposon mutants of *S. epidermidis* 1457-M10, M22, M23, M24 is shown in Figure 3. Inactivation of *icaA* in mutant 1457-M10 and of *icaC* in mutants M22 and M23 lead to biofilm negative and PIA negative phenotype, but did not alter 20-kDaPS antigen detection. The fact that mutant M24, where the transposon is oriented in the opposite transcriptional direction than the *icaADBC* operon and no *ica* specific transcript can be identified, still expressed 20-kDaPS provide clear proof that 20-kDaPS synthesis is independent of the *icaADBC* operon.

### Influence of chemical and enzymatic treatments on antigen detection by immunofluorescence and on biofilm integrity

**Figure 4:**
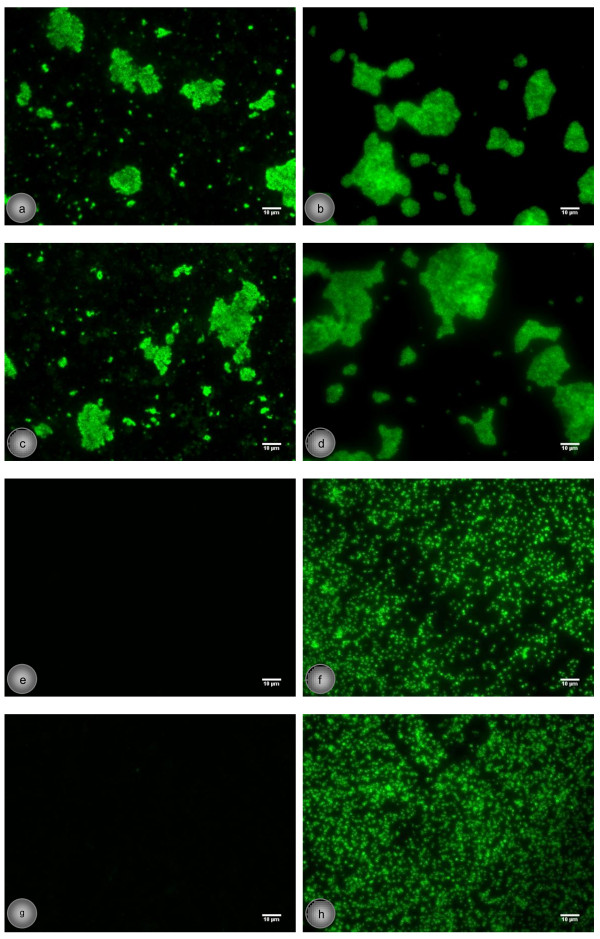
**Influence of proteinase K, periodate and DspB treatments on PIA and 20-kDaPS.** Immunofluorescence detection of PIA (**a**, **c**, **e**, **g**) and 20-kDaPS (**b**, **d**, **f**, **h**) on *S. epidermidis* 1457 grown as biofilm (**a**, **b**) after treatment with proteinase K (**c**, **d**), sodium *meta*-periodate (**e**, **f**) and DspB (**g**, **h**).

Periodate oxidation led to abolishment of antigenic reactivity of PIA, whereas 20-kDaPS preserved its antigenic properties (Figures 4e and 4f). Treatment with dispersin B (DspB) completely destroyed antigenic reactivity of PIA within one hour of incubation. DspB is a hexosaminidase (β-N-acetylglucosaminidase) produced by the oral pathogen *Aggregatibacter actinomycetemcomitans*, which specifically cleaves β-1,6-linked N-acetylglucosamine polymer disrupting PIA chain [38,39]. In contrast, DspB does not alter 20-kDaPS antigenic properties (Figures 4g and 4h). Parallel to PIA destruction, biofilm structure is disrupted after periodate oxidation and DspB treatments and large clumps are substituted by small clumps or single and double cells, still detectable by anti-20-kDaPS antiserum (Figure 4). Finally, the fact that PIA and 20-kDaPS retain their antigenic properties after proteinase K digestion is consistent with their polysaccharide nature (Figures 4c and 4d). Integrity of biofilm, formed on 96-well cell culture plates, to treatment with proteinase K, sodium *meta*-periodate and DspB was also studied. All biofilms were susceptible to sodium *meta*-periodate and DspB, whereas, addition of proteinase K did not affect biofilm stability. Thus, biofilm production in our strain collection is mediated mainly through PIA, as was shown in other studies [40-42]. In addition, 20-kDaPS presence does not relate to biofilm formation as agents, such as sodium *meta*-periodate and DspB that destroy biofilm integrity, do not affect antigenic properties of 20-kDaPS.

### Lack of co-purification of 20-kDaPS with PIA polysaccharide I in Q-Sepharose anion-exchange chromatography

**Figure 5:**
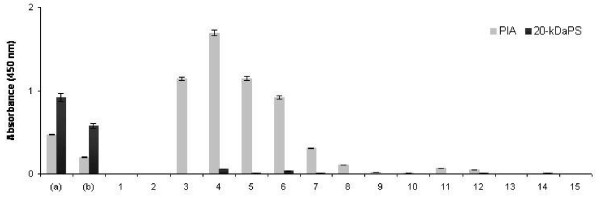
**PIA and 20-kDaPS detection in clarified bacterial extracts and Q-Sepharose eluted fractions.** PIA and 20-kDaPS detection in clarified bacterial extracts diluted 1:500 (a) and 1:2,000 (b) and Q-Sepharose column fractions (1–15) diluted 1:20. PIA and 20-kDaPS rabbit antisera were used at 1:800 and 1:3,000 dilutions, respectively. Presented data represent mean absorbance values ± SDs for two independent experiments performed in triplicate.

Clarified crude bacterial extracts obtained after bacterial sonication were tested for presence of PIA and 20-kDaPS reactivity by ELISA using anti-PIA and anti-20-kDaPS rabbit antisera, respectively (Figure 5). Under the conditions employed, in the crude extract consistently higher absorbance values were obtained with the 20-kDaPS specific antiserum as compared to the anti-PIA specific antiserum. The crude extract was applied to a Q-Sepharose column as described in Materials and Methods. Under these conditions the majority of PIA (approx. 80%) did not bind to the columns, but was immediately eluted. This PIA antigen fraction is referred to as polysaccharide I of PIA [4]. However, in the fractions representing the PIA antigenic peak reactivity with the specific anti-20-kDaPS antiserum was negligible indicating that 20-kDaPS does not co-purify with polysaccharide I of PIA. Additionally, this excludes significant cross reactivity of the 20-kDaPS antiserum with epitopes present on PIA.

### PIA and 20-kDaPS antisera do not cross-react with each-other

In order to identify any cross reactivity among 20-kDaPS antiserum and PIA antigen and *vice versa*, absorption studies were performed. PIA-specific antiserum was absorbed by *S. epidermidis* 1457 (PIA^+^ 20-kDaPS^+^) strain, as described in Methods. Absorbed antiserum was incubated with 1457 on immunofluorescence slides and achievement of complete absorption was confirmed. Furthermore, absorbed antiserum did not detect PIA on RP12 (PIA^+^ 20-kDaPS^+^), 1477 (PIA^+^ 20-kDaPS^+^) and 1510 (PIA^+^ 20-kDaPS^-^) *S. epidermidis* strains. PIA-specific antiserum was also absorbed by *S. epidermidis* 1510 (PIA^+^ 20-kDaPS^-^) and immunofluorescence tests performed with *S. epidermidis* RP12, 1457 and 1477. No remaining anti-PIA reactivity was observed with any strain using the absorbed antiserum. Finally, PIA-specific antiserum absorbed with *S. epidermidis* 1522 (PIA^-^ 20-kDaPS^+^) retains all reactivity to *S. epidermidis* 1457, RP12 and 1477 strains. In case that PIA antiserum reacted - even weakly - with 20-kDaPS antigen, incubation of PIA antiserum with strain 1522 bearing 20-kDaPS antigen, would lead to absorption of anti-PIA antibodies and no anti-PIA reactivity would remain. A selection of analogous experiments was performed regarding anti-20kDaPS serum, as shown in Table 1.

**Table 1:** Cross absorption experiment

	***anti-PIA serum absorbed by***				***anti-20 kDa PS serum absorbed by***			
	**1457**^a^	**1510**^a^	**1522**^a^		**1457-M10**^a^	**1522**^a^	**1510**^a^	**1505**^a^
	PIA^+^20kDaPS^+^	PIA^+^20kDaPS^-^	PIA^-^20kDaPS^+^		PIA^-^20kDaPS^+^	PIA^-^20kDaPS^+^	PIA^+^20kDaPS^-^	PIA^-^20kDaPS^-^
**1457**^b^	- ^c^	-	+ ^d^	**1457-M10**^b^	-	-	+	+
PIA^+^20kDaPS^+^				PIA^-^ 20kDaPS^+^				
**1510**^b^	-	-	*+*	**1457**^b^	-	-	+	+
PIA^+^20kDaPS^-^				PIA^+^20kDaPS^+^				
**RP12**^b^	-	-	*+*	**RP12**^b^	-	-	+	+
PIA^+^20kDaPS^+^				PIA^+^20kDaPS^+^				
**1477**^b^	-	-	+	**1522**^b^	-	-	+	+
PIA^+^20kDaPS^+^				PIA^-^20kDaPS^+^				
				**1477**^b^	-	-	+	+
				PIA^+^20kDaPS^+^				

^a^ Strains used for absorption of specific antisera, ^b^ Strains applied on immunofluorescence slide, ^c^ no fluorescence indicated no residual reactivity for specific antigen, ^d^ fluorescence indicated reactivity for specific antigen.

### Synthesis of 20-kDaPS and PIA in different culture media

In order to explore possible polysaccharide synthesis dependence on certain constituents of culture media, 20-kDaPS and PIA presence upon prolonged culture in different culture media was studied. 20-kDaPS expression was not abolished after long time incubation of bacteria in any of the selected media (RPMI1640, RPMI1640 + glutamine, IMDM, TSB, TSB w/o dextrose and on blood agar plates). 20-kDaPS antiserum revealed strong reactivity to bacterial cells growing in all media with the exception of TSB w/o dextrose where only a percentage of bacterial cells express 20-kDaPS. Regarding PIA synthesis, TSB seems superior to RPMI 1640, RPMI 1640 + glutamine and IMDM upon prolonged consecutive subcultures, whereas PIA expression was almost abolished in TSB lacking dextrose, in accordance to previous reports [7]. In addition, PIA presence was strongly associated to biofilm formation. Biofilms formed in RPMI1640, RPMI1640 + glutamine and IMDM were more susceptible to mechanic disruption following agitation by vortex and disintegration into small clumps (Table 2).

**Table 2:** Immunofluorescence upon prolonged culture in different chemically defined media

	**biofilm formation**	**anti-PIA**	**anti-20-kDaPS**		
	1457	1457	1457	1457-M10	RP12
**RPMI1640**	weak	+*	++	++	++
**RPMI1640 + Glutamine**	weak	+*	++	++	++
**IMDM**	weak	+*	++	++	++
**TSB**	strong	++	++	++	++
**TSB w/o Dextrose**	negative	-	+°	+°	+°
**Blood agar**		+*	++	++	++

* small clumps, ° few cells, ++ strong fluorescence, - no fluorescence.

### Impact of 20-kDaPS on bacterial endocytosis

**Figure 6:**
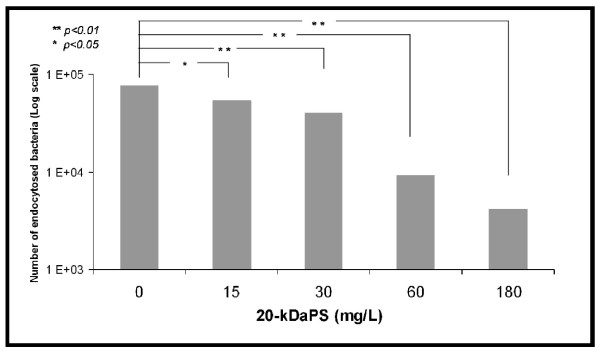
**Impact of 20-kDaPS on endocytosis of*S. epidermidis*by human macrophages.** Bacterial suspensions of non-20-kDaPS producing *S. epidermidis* clinical strain, preincubated with different concentrations of 20-kDaPS, were added to human macrophages. The number of endocytosed bacteria was counted by serial dilutions of cell lysates on blood agar. All experiments were repeated five times.

**Figure 7:**
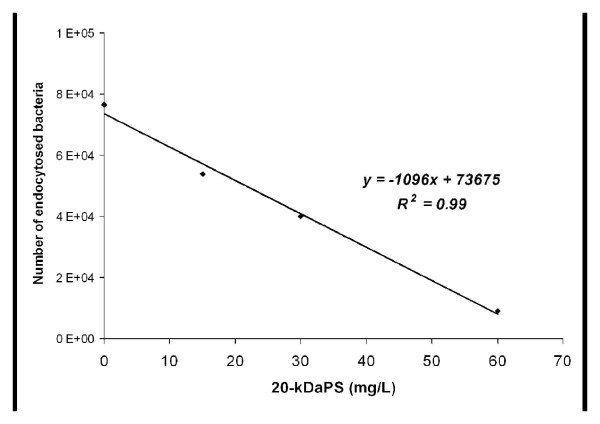
**20-kDaPS inhibits endocytosis of*S. epidermidis*in a dose-dependent manner.** Standard curve obtained by counting the number of endocytosed bacteria preincubating with increasing amounts of 20-kDaPS (0, 15, 30, 60 mg/L) (y = −1096x + 73675, *R*^*2*^ = 0.99.

**Figure 8:**
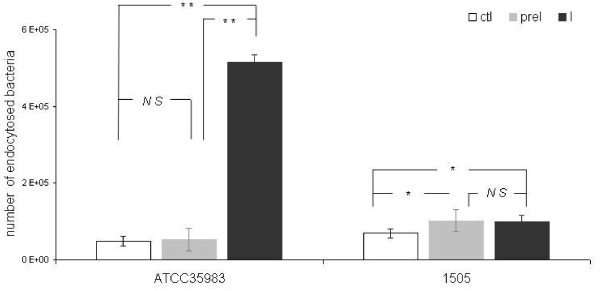
**Impact of 20-kDaPS antiserum on endocytosis of*S. epidermidis*by human macrophages.** Bacterial suspensions of 20-kDaPS-producing *S. epidermidis* reference strain ATCC35983 and non-20-kDaPS producing *S. epidermidis* clinical strain 1505 preincubated with PBS (ctl), preimmune serum (preI), and 20-kDaPS antiserum (I) were added to human macrophages. The number of endocytosed bacteria was counted by serial dilutions of cell lysates on blood agar. Columns represent mean values of endocytosed bacteria from a representative experiment out of five similar ones performed in triplicate. (*) *p* < 0.05, (**) *p* < 0.005, (NS) *p* > 0.05.

Differences in phagocytosis between *S*. *epidermidis* reference strain ATCC35983 and the clinical 20-kDaPS negative strain 1505 were observed (48,300 ± 2,400 cfu *vs* 68,800 ± 4,700 cfu, respectively, *p < 0.05*). Phagocytosis experiments were performed without addition of exogenous complement. Preincubation of non-20kDaPS-producing strain with different concentrations of 20-kDaPS inhibits endocytosis (Figure 6). Specifically, preincubation of non-20kDaPS-producing strain with 20-kDaPS (0, 15, 30, 60, 180 μg/mL) reduces the number of endocytosed bacteria from 76,500 ± 7,400 to 54,000 ± 1,300, 40,000 ± 2,271, 9,100 ± 2,193, 4,100 ± 793 bacteria/well, respectively. Differences are statistically significant in all above 20-kDaPS concentrations.Inhibition of endocytosis takes place at a dose dependent manner between 0 and 60 μg/mL (Figure 7). On the contrary, 20-kDaPS antiserum increases endocytosis of 20-kDaPS-producing ATCC35983 strain *ca* 10 fold, as compared to bacteria preincubated with preimmune serum (516,800 ± 52,500 cfu *vs* 52,800 ± 28,800, *p < 0.005*). Preincubation with preimmune antiserum did not alter endocytosis, as compared to bacteria preincubated with PBS (48,300 ± 2,400 cfu vs 52,800 ± 28,800 cfu). In terms of *S*. *epidermidis* clinical isolate 1505, preincubation with preimmune antiserum seems to enhance endocytosis, as compared to bacteria preincubated with PBS (101,600 ± 10,400 *vs* 68,800 ± 8,700 cfu, respectively, *p < 0.05*), but preincubation with 20-kDaPS antiserum does not further increase endocytosis, as compared to bacteria preincubated with preimmune serum (98,300 ± 17,900 cfu *vs* 101,600 ± 10,400 cfu, *p > 0.05*). This phenomenon may be associated with the presence of other anti-staphylococcal antibodies in rabbit serum. Prior to immunization, rabbit serum was collected and tested by ELISA for reactivity to 20-kDaPS in order to exclude pre-existence of 20-kDaPS specific antibodies. Low titers of antibodies to various staphylococcal strains, *S. epidermidis* and *S. aureus*, are present in preimmune serum (data not shown) and may be responsible for the observed effect. A representative experiment of five similar ones is presented in Figure 8.

## Discussion

*Staphylococcus epidermidis* is an important pathogen [43] and extracellular polysaccharides as well as a number of surface proteins contributing to bacterial attachment and biofilm formation have been extensively studied. Analysis of *S. epidermidis*’ polysaccharides has been associated with difficulties, however, it is now clear that, despite some possible variation, PIA, and other analogue polysaccharides such as PS/A, PNSG, PNAG, and SAA are chemically closely related if not identical and represent the same chemical entity, namely PIA. This is the first time shown that 20-kDaPS is discrete from PIA and this statement is based on concrete basis.

Transposon insertion in *icaADBC*, the locus encoding synthetic enzymes for PIA synthesis, does not abrogate production of 20-kDaPS. In mutant 1457-M10 in which Tn*917* was inserted in *icaA* in the same transcriptional orientation, outward directed transcription resulted in transcripts comprising the complete sequences of *icaDicaB* and *icaC*[44]. Expression of 20-kDaPS in mutant 1457-M10 where *icaA* synthesis is inhibited and in mutant M22 and M3 where *icaC* expression was inhibited shows that 20-kDaPS synthesis does not require an intact *icaA* or *icaC* gene. The fact that 20-kDaPS was detected in M24, where Tn*917* was inserted in the opposite transcriptional direction to the *ica* operon and no-*ica* specific transcripts were identified [44], provides evidence that 20-kDaPS synthesis is independent of *ica* operon. In contrast, PIA synthesis is completely inhibited not only by the disruption of the entire *icaADBC* operon but also by the isolated inhibition of *icaA* (M10) and *icaC* (M22, M23) gene expression.

Proteinase K does not disrupt antigenic properties of 20-kDaPS reconfirming its polysaccharide nature. Furthermore, DspB, which specifically cleaves β-1,6-linked N-acetylglucosamine polymer disrupting PIA chain [38,39], did not affect 20-kDaPS. Although sodium *meta*-periodate is an agent commonly used to disrupt polysaccharide molecules, it did not affect integrity of 20-kDaPS antigen. Taking into account that periodate preferably degrades *cis*-diols, it is suggested that monomeric units of the polysaccharide core form glycosidic bonds between the anomeric C-1 and the C-3 or C-4. This is not the case for PIA, where a β-1,6-glycosidic bond is present leaving free vicinal hydroxyl groups of glucosamine at C-3 and C-4. The above structural data suggest that 20-kDa PS and PIA are two discrete and different polysaccharides. Preliminary data in our laboratories showed that 20-kDaPS is not affected upon treatment with glycosaminoglycan- degrading enzymes (heparin lyases, keratanases and chondroitinases), suggesting a non glycosaminoglycan-related structure.

Absence of 20-kDaPS in Q-Sepharose fractions containing maximum PIA reactivity is due to different physicochemical properties among the two molecules. Q-Sepharose is a strong anion-exchanger which retains negatively charged molecules. Whereas PIA is eluting, 20-kDaPS may be strongly retained by the column due to its negative charges. Aforementioned differentiation was expected as different isolation procedures are used for the two polysaccharides. As previously described [16,19], 20-kDaPS is obtained from bacterial extracellular matrix using a linear NaCl gradient on DEAE-Sephacel and elutes at 0.5-0.7 M NaCl.

Presented data suggest that 20-kDaPS inhibits endocytosis of *S*. *epidermidis* bacterial cells at a dose-dependent manner. Similarly, PIA provides protection against opsonophagocytosis and activity of anti-microbial peptides [9,10]. In the absence of specific opsonizing antibodies, macrophages are able to clear pathogens by innate immune receptors, such as the group of molecular pattern recognition receptors (PRR), collectively known as scavenger receptors [45]. 20-kDaPS may interfere with or mask staphylococcal antigen(s) promoting phagocytosis [46]; on the other hand, it may interact with a receptor that does not facilitate phagocytosis. Adhesion receptors and phagocytosis receptors can both activate and inhibit each other functions [47]. It has been previously shown that 20-kDaPS promotes adhesion to human endothelial cells and this interaction is blocked upon addition of anti-20kDaPS antibodies. Comparable data were acquired by using human macrophages (data not shown), indicating the presence of a specific ligand for 20-kDaPS on human cells. Adherence of unopsonized bacteria to macrophages does not preclude internalization [48-51]. Nonopsonic binding of pathogens to host phagocytic cells may not always result in phagocytosis, however, it may serve an important role in the immune response [52]

Nevertheless, phagocytic activity of macrophages is greatly enhanced if specific antibodies are attached to the pathogen [53]. 20-kDaPS antiserum do not exhibit any cross reactivity with PIA. Antibodies against PNSG and PIA have been found completely cross-reactive [31]. As 20-kDaPS antiserum reacts specifically and strictly with 20-kDaPS, observed biologic properties concern exclusively this entity. Our data show that 20-kDaPS antiserum exhibits opsonic properties as it increases endocytosis of *S. epidermidis* ATCC35983 by human macrophages. Several surface molecules have been studied as potential antibody targets in order to enhance phagocytic potential of monocytes/macrophages. Opsonic activity of antibodies to *S. epidermidis* Fbe and AtlE has been demonstrated in a study where fresh alveolar macrophages from rat ingested and killed *S. epidermidis* opsonized with anti-Fbe antibodies (raised in rabbit, rat or sheep) to a much higher extent than they ingested and killed nonopsonized bacteria or bacteria opsonized with antibodies directed against AtlE or Embp [53]. Also, a chimerized (murine/human) monoclonal antibody against lipoteichoic acid that was proven protective for CoNS and *S. aureus* bacteremia in animal models has been also tested to humans [54]. In contrast, antibodies to accumulation-associated protein and lipoteichoic acid had no opsonic activity *in vitro* and did not protect mice against experimental biomaterial-associated infections [55]. Although, conjugate vaccines based on PIA/PNAG have been shown to be beneficial in animal models [56-60], several doubts for their use in human trials have been documented [61,62]. Thus, more and extensive investigations are needed to evaluate the potential use of 20-kDaPS in conjugate vaccines.

## Conclusions

This is the first study providing concrete data that 20-kDaPS is a unique polysaccharide molecule discrete from PIA. 20-kDaPS exhibits antiphagocytic properties that may be shown to play a role in pathogenicity. Further work is in progress to establish a role in conjugate vaccine development.

## Methods

### Bacterial strains

Two reference *S. epidermidis* strains, ATCC35983 (RP12) and ATCC35984 (RP62A) were used in the present study. Biofilm-producing, PIA-positive *S. epidermidis* strains 1457, 9142, 8400, and isogenic biofilm-negative, PIA-negative transposon mutants 1457-M10, M22, M23, M24 and 8400-M10 with Tn*917* insertion in the *icaADBC* operon have been described. In mutants 1457-M10 and M24, Tn*917* inserted in *icaA* whereas in M22 and M23 the transposon inserted in *icaC*[6,7,31,42,63]. The transposon was oriented in the same transcriptional direction as the *icaADBC* operon in all mutants except for M24 in which the transposon inserted in the opposite direction. Also, biofilm-negative, PIA-negative *S. epidermidis* strains 5179 and 1585 as well as biofilm-positive, PIA-negative variant 5179-R1 were used [7,64,65] (see also Table 3).

**Table 3:** ***S. epidermidis*reference and clinical strains used in the present study**

*S. epidermidis* strains				
1457	biofilm^+^PIA^+^	*ica*^+^	20-kDaPS^+^	Mack *et al.*, 1992
1457-M10	biofilm^-^PIA^-^	*icaA*::Tn*917*	20-kDaPS^+^	Mack *et al.*, 1994
M22	biofilm^-^PIA^-^	*icaC*::Tn*917*	20-kDaPS^+^	Mack *et al.*, 2000
M23	biofilm^-^PIA^-^	*icaC*::Tn*917*	20-kDaPS^+^	Mack *et al.*, 2000
M24	biofilm^-^PIA^-^	*icaA*::Tn*917*	20-kDaPS^+^	Mack *et al.*, 2000
8400	biofilm^+^PIA^+^	*ica*^+^	20-kDaPS^+^	Mack *et al.*, 1992
8400-M10	biofilm^-^PIA^-^	*icaA*::Tn*917*	20-kDaPS^+^	Mack *et al.*, 1999
9142	biofilm^+^PIA^+^	*ica*^+^	20-kDaPS^+^	Mack *et al.*, 1992
5179	biofilm^-^PIA^-^	*icaA*::*IS257*	20-kDaPS^+^	Mack *et al.*, 1992
5179R1	biofilm^+^PIA^-^	*icaA*::*IS257 aap*^+^	20-kDaPS^+^	Rohde *et al.*, 2005
1585	biofilm-PIA^-^	*ica*-	20-kDaPS-	Rohde *et al.*, 2005
ATCC35983 (RP12)	biofilm^+^PIA^+^	*ica*^+^	20-kDaPS^+^	Reference strain
ATCC35984(RP62A)	biofilm^+^PIA^+^	*ica*^+^	20-kDaPS^+^	Reference strain
1477	biofilm^+^PIA^+^	*ica*^+^	20-kDaPS^+^	Clinical strain.
1522	biofilm^-^PIA^-^	*ica*-	20-kDaPS^+^	Clinical strain
1510	biofilm^+^PIA^-^	*ica*^+^	20-kDaPS-	Clinical strain
1505	biofilm^-^PIA^-^	*ica*-	20-kDaPS-	Clinical strain

Seventy-five clinical CoNS isolates from blood cultures and central venous catheter tips collected in the Clinical Laboratory of General University Hospital of Patras, Greece, were used in the present study (50 *S. epidermidis*, 12 *S. haemolyticus*, 9 *S. hominis*, 1 *S. cohnii*, 1 *S. xylosus*, 1 *S. capitis*, 1 *S. lugdunensis*). Clinical strains were identified at the species level (API Staph ID 32 cards and automated VITEK system, BioMerieux) and tested for the presence of *icaAicaD1icaD2icaC* by PCR [66-68]. Ability of clinical strains for biofilm formation was assessed quantitatively on microtiter plates, as previously described [7,69,70].

### Antisera

Specific PIA antiserum raised in rabbits against purified polysaccharide I of PIA and specific 20-kDaPS antiserum raised in rabbits against purified 20-kDaPS has been previously described [4,19,70].

### Specific antigen detection by immunofluorescence

Detection of 20-kDaPS and PIA by immunofluorescence was performed, as previously described [7,70]. Briefly, overnight cultures of *S. epidermidis* strains in TSB were diluted 1:100 in 2 mL fresh medium and incubated for 18 h at 37°C with shaking. After brief vortex, bacterial suspensions were adjusted to approximate absorbance_578_ 0.2 (Spectrophotometer, Novaspec Plus) and aliquots (10 μL per well) were applied to immunofluorescence slides (CA Hendley Essex Ltd, Essex, United Kingdom). Slide preparations were air-dried, fixed with cold acetone and stored at 4°C until use. Aliquots (20 μL per field) PIA or 20-kDaPS antisera diluted 1:50 in PBS were applied to slides which were incubated for 30 min at 37°C. After washing three times with PBS, 10 μL of fluorescein-conjugated anti-rabbit immunoglobulin G (Sigma, UK) diluted 1:80 in phosphate buffered saline were applied, and slides were incubated for 30 min at 37°C. After washing, they were mounted using Vectashield and viewed with a Zeiss AxioImager fluorescence microscope fitted with an AxioCam MR3 camera.

### Specific antigen detection by ELISA

ELISA for polysaccharide detection was performed as previously described [17]. Briefly, antigens, bacterial cells or polysaccharide, were applied on a 96-well flat bottom high binding ELISA plate (Greiner) and incubated overnight at 4°C. Afterwards, plates were blocked by BSA and incubated with 20-kDaPS or PIA antisera for 1 h at 37°C. Peroxidase H-conjugated goat anti-rabbit IgG (Sigma Chemical Company, St Louis, MO, USA), diluted 1:2,000 was added for 1 h. Color was developed by adding 100 μL/well SureBlue TMB Microwell Peroxidase Substrate (KPL). After incubation for 15 min at room temperature in the absence of light, the reaction was terminated with 100 μL/well of 1 M H_2_SO_4_ and measured at absorbance_450_. ELISA was also performed, as previously described, on 96-well tissue culture plates (Nunc) with similar results.

### PIA isolation

Isolation of PIA antigen was performed, as previously described [6], with slight modification. Briefly, *S. epidermidis* 1457 was grown for 22 h at 37°C with shaking at 100 rpm/min in 900 mL of TSBdia, prepared by dialysis of 100 mL of 10-fold-concentrated TSB against 900 mL of water. Bacterial cells were collected by centrifugation and were suspended in 20 mL of PBS. The antigen was extracted by sonicating cells four times for 30 sec on ice (Branson Digital Sonifier). Cells were removed by centrifugation at 6,000 rpm for 30 min at 4°C, and extracts were clarified by centrifugation for 60 min at 12,000 rpm. The extracts (20 mL) were filter sterilized, dialyzed against 50 mM Tris–HCl, pH 7.5, overnight, concentrated by using Centriprep 10 (Amicon, Witten), applied to PD-10 Q-Sepharose column (Sigma) equilibrated with 50 mM Tris–HCl, pH 7.5, and fractions of 1.5 mL were collected.

### Influence of proteinase K, sodium *meta*-periodate and dispersin B treatments on antigen integrity and biofilm stability

Overnight cultures of different *S. epidermidis* strains in TSB were diluted 1:100 in 5 mL fresh TSB and incubated in 6-well flat-bottom tissue culture plates (Nunc) for additional 16–18 h at 37°C. Supernatants were removed and biofilms were detached using a cell scraper and suspended in 2 mL PBS. After brief vortex bacterial suspensions were adjusted to absorbance_578_ 0.2. Aliquots of bacterial cultures (200 μL) were supplemented with 40 μL of 0.2 M sodium *meta*-periodate (Sigma), 2 μL of 100 μg/mL proteinase K (Promega, Madison, WI, USA), 2 μL of 1 mg/mL DspB and incubated at 4°C for 16 h, 37°C for 16 h and 37°C for 1 h and 5 h, respectively. Samples were applied onto immunofluorescence slides at appropriate dilution and immunofluorescence tests performed as described above. For testing the stability of established biofilms, bacteria were grown overnight in 96-well cell tissue culture plates (Nunc) as described above. Medium was removed and PBS containing proteinase K (1 μg/mL) or DspB (10 μg/mL) or sodium *meta*-periodate (0.04 M) was added for 16 h at 37°C and at 4°C for sodium *meta*-periodate. Disruption of biofilm integrity was evaluated by assessment the absorbance at 570 nm.

### Absorption of antiserum

20-kDaPS and PIA antiserum were absorbed, as previously described [7], with slight modification. In brief, overnight cultures of selected strains were diluted 1:100 in TSB and incubated with shaking at 100 rpm for 18 h. Bacteria were harvested, washed two times in PBS and resuspended in PBS (absorbance_578_ =2). Aliquots of this bacterial preparation (50 μL) were incubated with one μL of the respective antiserum diluted in 450 μL PBS overnight at 4°C on a rotating wheel. Bacterial cells were removed by centrifuging twice at 12,000 × *g* for 15 min in a mini-centrifuge and the supernatants were filter sterilized.

### Antigen expression upon bacterial culture in chemically defined media

*S. epidermidis* strains 1457, 1457-M10, and RP12 were subcultured daily for ten days in the following chemically defined broth media: RPMI1640, RPMI1640 + glutamine, IMDM, (Gibco, Invitrogen Life Science), TSB, TSB w/o dextrose and on blood agar plates. 20-kDaPS and PIA expression was assessed by immunofluorescence on day 1, 4, 7 and 10.

### Human monocyte derived macrophages

Human peripheral blood mononuclear cells were isolated from buffy coats by density centrifugation on Ficoll density gradient (Biochrom AG, Berlin) and incubated for 2 h in RPMI-1640 medium supplemented with 10% heat-inactivated FCS (Biochrom AG, Berlin) and 2 mM L-Glutamine (HyClone) in 75 cm^2^ tissue culture flasks (Sarstedt Inc, Newton, NC, USA) at 37^o^ C in a humidified, 5% CO_2_ atmosphere. Afterwards, non adherent cells were discarded and adherent cells were collected with a cell scraper. Monocytes were differentiated to macrophages after 7 days culture in RPMI-1640 medium supplemented by Gentamicin, Penicillin-Streptomycin (Gibco, Invitrogen, Grand Island, NY, USA), 10% heat-inactivated human AB serum (Invitrogen, USA), 2 mM L-Glutamine and macrophage colony-stimulating factor (10 ng/mL; Abcam, UK). Experimental work using human blood mononuclear cells carried out after obtaining written informed consent of healthy blood donors and was approved by the University of Patras Bioethics Committee.

### Bacterial endocytosis

In order to assess the impact of 20-kDaPS on *S*. *epidermidis* endocytosis, one hundred microliters of a non-20-kDaPS-producing clinical strain (strain 1505) (2 × 10^8^ bacteria/mL) were incubated at room temperature with increasing concentrations (0, 15, 30, 60 μg/mL) of 20-kDaPS. In order to assess the impact of 20-kDaPS antiserum on *S*. *epidermidis* endocytosis, 100 μL of 20-kDaPS-producing strain ATCC35983 and 100 μL of non-20-kDaPS-producing clinical strain (2 × 10^8^ bacteria/mL) were incubated at room temperature with PBS, preimmune antiserum and 20-kDaPS antiserum for one h. Afterwards, bacterial suspensions were centrifuged at 12000 × *g* for ten minutes and further washed with PBS. This procedure was repeated three times. Finally, bacteria were resuspended in PBS at final concentration of 2 × 10^7^ bacteria/mL. Two hundred thousand (2 × 10^5^) macrophages in 0.5 mL RPMI1640 were incubated with 2 × 10^6^ bacteria preincubated with 20-kDaPS in different concentrations, preimmune antiserum, 20-kDaPS antiserum or PBS at 37°C for one h. Then, 10 μL lysostaphin (1 mg/mL) was added for 15 min and cells were washed with PBS. Absence of live extracellular bacteria was confirmed by absence of growth on blood agar. Cells were lysed by 0.1% Triton X-100 and viable intracellular bacteria were counted by plating serial dilutions of the lysates on blood agar plates. Experiments were performed at least five times in triplicate using macrophages from different donors.

### Statistical analysis

Statistical analysis was performed using SPSS 17 statistical package (SPSS Inc, USA). Differences were evaluated using paired *t* test.

## Authors’ contributions

**AS** carried out experimental work and drafted the manuscript. **FK** designed and participated in experiments involving analysis of clinical strains. **MK** participated in experiments for 20-kDaPS isolation and helped to draft the manuscript. **LH** participated in experiments involving comparison of PIA and 20-kDaPS by immunofluorescence and contributed to design of these experiments. **TW** participated in experiments involving comparison of PIA and 20-kDaPS by ELISA and contributed to design of these experiments. **AD** participated in the design of the study. **GD** contributed to design of phagocytosis experiments. **NK** contributed to design of phagocytosis experiments, structural elucidation, data interpretation and revised the manuscript. **DM** designed the study and experimental work involving comparison of PIA and 20-kDaPS, interpreted acquired data and revised the manuscript. **EA** conceived of the study, participated in its design and interpretation of acquired data and revised the manuscript. All authors read and approved the final manuscript.
